# The volatile emission of *Eurosta solidaginis* primes herbivore-induced volatile production in *Solidago altissima* and does not directly deter insect feeding

**DOI:** 10.1186/1471-2229-14-173

**Published:** 2014-06-19

**Authors:** Anjel M Helms, Consuelo M De Moraes, Mark C Mescher, John F Tooker

**Affiliations:** 1 Department of Entomology, Center for Chemical Ecology, The Pennsylvania State University, University Park, USA; 2 Department of Environmental Systems Science, ETH Zürich, Zürich, Switzerland

**Keywords:** *Solidago altissima*, *Eurosta solidaginis*, Priming, Herbivore-induced plant volatiles

## Abstract

**Background:**

The induction of plant defenses in response to herbivory is well documented. In addition, many plants prime their anti-herbivore defenses following exposure to environmental cues associated with increased risk of subsequent attack, including induced volatile emissions from herbivore-damaged plant tissues. Recently, we showed in both field and laboratory settings that tall goldenrod plants (*Solidago altissima*) exposed to the putative sex attractant of a specialist gall-inducing fly (*Eurosta solidaginis*) experienced less herbivory than unexposed plants. Furthermore, we observed stronger induction of the defense phytohormone jasmonic acid in exposed plants compared to controls. These findings document a novel class of plant-insect interactions mediated by the direct perception, by plants, of insect-derived olfactory cues. However, our previous study did not exclude the possibility that the fly emission (or its residue) might also deter insect feeding via direct effects on the herbivores.

**Results:**

Here we show that the *E. solidaginis* emission does not (directly) deter herbivore feeding on *Cucurbita pepo* or *Symphyotrichum lateriflorum* plants*—*which have no co-evolutionary relationship with *E. solidaginis* and thus are not expected to exhibit priming responses to the fly emission. We also document stronger induction of herbivore-induced plant volatiles (HIPV) in *S. altissima* plants given previous exposure to the fly emission relative to unexposed controls. No similar effect was observed in maize plants (*Zea mays*), which have no co-evolutionary relationship with *E. solidaginis.*

**Conclusions:**

Together with our previous findings, these results provide compelling evidence that reduced herbivory on *S. altissima* plants exposed to the emission of male *E. solidaginis* reflects an evolved plant response to olfactory cues associated with its specialist herbivore and does not involve direct effects of the fly emission on herbivore feeding behavior. We further discuss mechanisms by which the priming of HIPV responses documented here might contribute to enhanced *S. altissima* defense against galling.

## Background

Despite their sedentary lifestyles, plants actively perceive and respond to a wide range of environmental cues, including those associated with attack by insect herbivores. Induction of plant defenses following insect herbivory is well characterized [1-4]. And recent work has shown that, prior to the onset of feeding, many plant species also express or prime anti-herbivore defenses in response to herbivore-associated environmental cues, including both physical and biochemical cues related to the physical presence of herbivores or their eggs [5-7]. Defense priming has furthermore been shown to occur in response to airborne chemical cues, specifically damage-induced volatile organic compounds emitted by neighboring plants (or distant parts of the same plant) that are already experiencing herbivory [8-14].

Recently, we documented an apparent example of similar defense priming in goldenrod plants (*Solidago altissima*) exposed to an olfactory cue derived directly from an insect herbivore—the putative sex pheromone of the specialist gall-inducing fly *Eurosta solidaginis*[15]. Specifically, we observed dramatically reduced herbivory—in both laboratory and field studies—on plants exposed to the volatile emission of male flies, as well as enhanced induction of the key defense phytohormone jasmonic acid in emission-exposed plants subjected to insect feeding damage. Building upon this work, the current study elucidates additional effects of exposure to the fly emission on *S. altissima* defense responses, as well as the direct effects of the emission itself on insect feeding.

It is well established that plants can respond to airborne chemicals. For example, the diverse and critical functions of the gaseous phytohormone ethylene have been documented and elucidated over many decades [16-19]. And numerous recent studies have elucidated the responsiveness of plants to environmentally derived olfactory cues. Parasitic plants in the genus *Cuscuta*, for example, have been shown to grow toward host-plant-derived volatiles [20], and, as noted above, plants can respond to plant odors elicited by insect feeding [9-13,21-23].

Our demonstration of *S. altissima* responses to the putative sex attractant of *E. solidaginis*[15] documented a novel class of plant-insect interactions mediated by plant perception of olfactory cues deriving directly from insect antagonists. In that study, we also proposed two alternative hypotheses that might influence the interpretation of our findings: (i) that the effects observed might reflect a biochemical manipulation of the host plant by the fly (rather than an adaptive plant response to a cue indicating the presence of the fly), and (ii) that some residue of the fly emission present on plant tissues might itself deter subsequent herbivory. The first of these hypotheses is difficult to reconcile with our previous finding that female *E. solidaginis* discriminate against emission-exposed plants in the field, which strongly suggests that the quality of these plants as hosts for fly offspring was compromised rather than enhanced [15]. The second alternative hypothesis is also countered by our previous findings, specifically the observation of significantly enhanced JA responses of exposed plants to subsequent herbivory, indicating that the observed effects are indeed mediated by physiological responses of the plant to exposure. However, the existence of such enhancement does not exclude the possibility that the fly emission might also have directly deterrent effects on insect feeding that contribute to the subsequent reduction in herbivory. The current study therefore sought to provide additional evidence that exposure to the *E. solidaginis* emission induces changes in *S. altissima* defense chemistry and to directly test the influence of the emission on feeding by insects.

To further explore *S. altissima* defense responses, we analyzed the volatile production of *S. altissima* plants exposed to the *E. solidaginis* emission and unexposed controls, both before and after herbivore damage. In addition to providing olfactory cues for neighboring plants, as discussed above, herbivore-induced changes in plant volatile emissions are thought to confer defensive benefits by providing cues that recruit natural enemies of feeding herbivores [24-29] or deter feeding or oviposition by additional herbivores [30-33]. Furthermore, volatile induction is known to be mediated by JA [34,35] and thus is likely to reflect downstream influences of the JA induction we documented previously. In addition to examining the effects of the *E. solidaginis* emission on *S. altissima* volatile responses, we conducted parallel experiments in maize (*Z. mays*). Because maize has no apparent co-evolutionary or ecological relationship with *E. solidaginis* and we did not previously observe reduced insect feeding on exposed maize [15], we predicted that exposure to the *E. solidaginis* emission would not induce changes in volatile induction in this plant species.

To explore potential direct effects of the *E. solidaginis* emission on insect feeding we performed feeding assays using striped cucumber beetles (*Acalymma vittatum*) feeding on *E. solidaginis* emission-exposed squash plants (*Cucurbita pepo* var. *texana*) or on unexposed controls and performed similar assays using goldenrod leaf beetles (*Trirhabda virgata*) feeding on emission-exposed calico aster (*Symphyotrichum lateriflorum*) or controls. Again because of the absence of any apparent association between *E. solidaginis* flies and squash or calico aster plants we did not expect squash or calico aster to exhibit any physiological response to the *E. solidaginis* emission, so that any reduction in feeding damage observed could likely be attributed to the direct deterrent effect of the emission.

## Results

### Volatile collections

**Figure 1:**
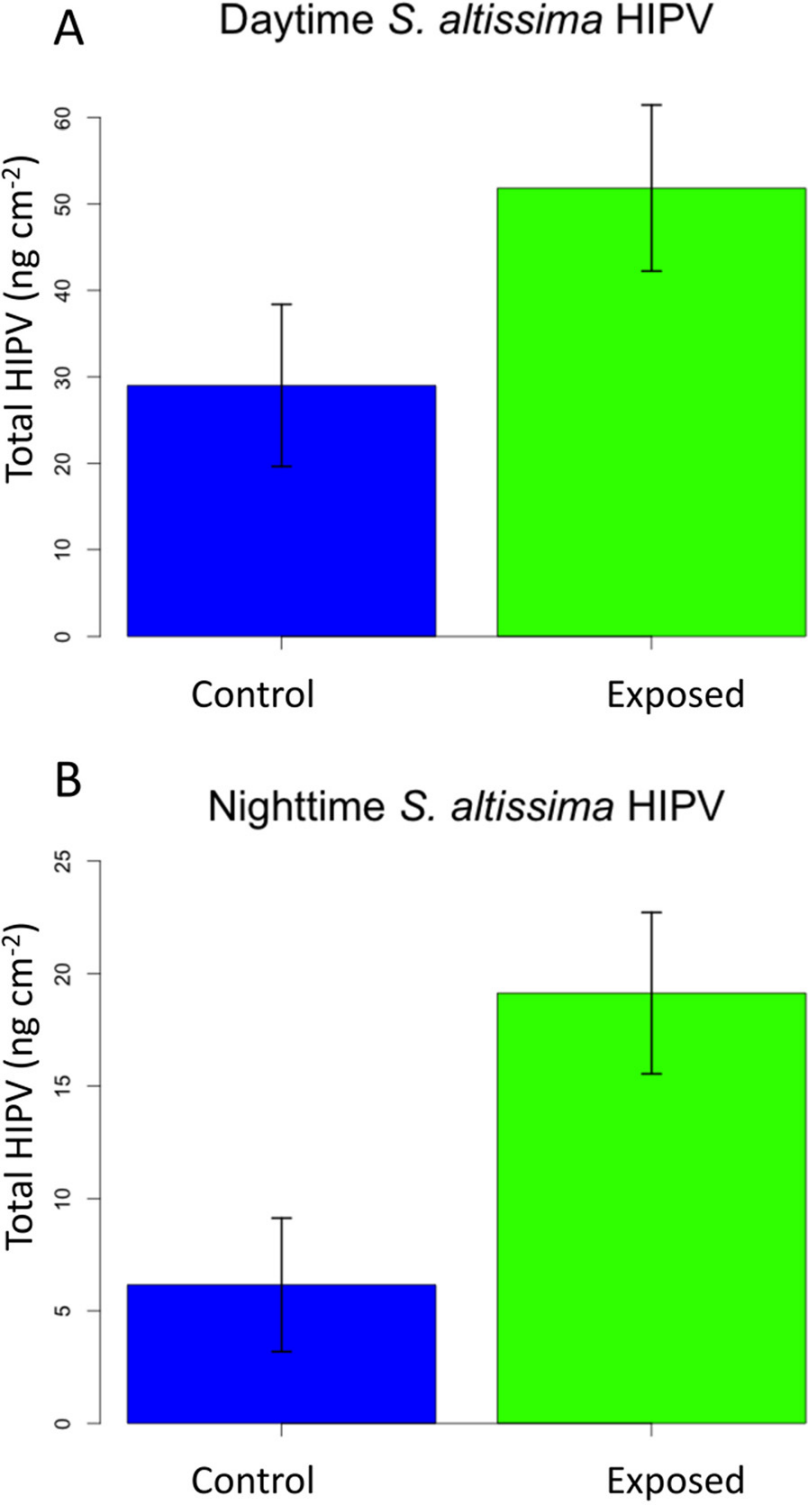
***Solidago altissima *herbivore-induced volatiles. (A)** Total herbivore-induced volatiles emitted by *S. altissima* plants exposed to the *E. solidaginis* emission and unexposed controls during 18 h photophase. **(B)** Total herbivore-induced volatiles emitted by *S. altissima* plants exposed to the *E. solidaginis* emission and unexposed controls during 6 h scotophase. Data are shown untransformed, but statistical analyses were performed on square-root transformed data.

To determine whether exposure to the *E. solidaginis* emission primed herbivore-induced volatile production in *S. altissima* plants, we analyzed the volatiles produced by *S. altissima* plants exposed to the emission and unexposed plants both before and after feeding damage by *Heliothis virescens* caterpillars. This generalist caterpillar species was used in place of *E. solidaginis* for the volatile-induction assays because it triggers a strong volatile response from *S. altissima* and because the galling habit of the flies makes them difficult to use for such assays [36]. Furthermore, by substituting a generalist leaf-chewing herbivore, we were able to compare volatile-induction by the same herbivore in both maize and goldenrod. Prior to herbivory, we found no difference in volatile production between *S. altissima* plants previously exposed to the emission and unexposed control plants (Additional file 1: Table S1). After feeding by *H. virescens* caterpillars, however, we found that the emission-exposed plants produced a greater total amount of herbivore-induced plant volatiles (HIPV) both during the day (29.0 ng cm^−2^ and 51.8 ng cm^−2^ for unexposed and exposed plants, respectively) and at night (6.2 ng cm^−2^ and 19.1 ng cm^−2^), indicating a more vigorous response to insect damage (Figure 1A, Day: two-sided *t*-Test, *t* = −1.93, df = 18, *P* = 0.069; Figure 1B, Night: two-sided *t*-Test, *t* = −3.00, df = 18, *P* = 0.0078). We collected volatiles during both the photophase and scotophase because previous studies documented substantial variation in volatile blends emitted during these phases and day- or night-active insects can be more responsive to the volatiles emitted during their times of peak activity [31,33].

We also conducted principal component analyses for both the daytime and nighttime HIPV and plotted the first two components from each to visualize which compounds in the blends might be driving the differences between treatments (Additional file 2: Figure S1A, S1B). For daytime HIPV, the first two principal components account for 96.3% of the variance. For nighttime HIPV, the first two principal components account for 96.4% of the variance. In total, we measured and identified twenty-three compounds in the *S. altissima* volatile blend. We found no novel compounds in the HIPV blend of emission-exposed plants compared to the control or when comparing the blends of damaged and undamaged plants; however, we identified a few specific compounds in the daytime and nighttime blends that were emitted in significantly greater amounts by the induced emission-exposed plants (Table 1). The compounds emitted in significantly greater amounts (*P* ≤ 0.05) in the daytime blend were the monoterpenes α-pinene, β-pinene, and limonene. Three compounds were also marginally significant, including bornyl acetate (*P* = 0.09) and the monoterpenes camphene (*P* = 0.09) and myrcene (*P* = 0.06). In the nighttime blend, emission-exposed *S. altissima* emitted the following compounds in significantly higher amounts (*P* ≤ 0.05) after herbivore damage: the monoterpenes α-pinene, β-pinene, myrcene, and limonene, the sesquiterpenes caryophyllene, α-humulene, β-farnescene, and germacrene D, and the compound bornyl acetate. Four compounds were marginally significant, including the green-leaf volatiles (GLV) (*Z*)-3-hexen-1-ol (*P* = 0.07) and (*Z*)-3-hexenyl isobutyrate (*P* = 0.07), the terpene alcohol linalool (*P* = 0.09) and (*Z*)-jasmone (*P* = 0.06). No compounds were emitted in significantly higher amounts by the unexposed control plants.

**Table 1:** **Day and night *Solidago altissima *** **individual herbivore-induced volatile organic compounds (VOC; means ± standard error; untransformed data shown)**

	**Daytime HIPV**			**Nighttime HIPV**		
	**Exposure treatment**					
	***Eurosta***	**Control**		***Eurosta***	**Control**	
**Compounds in *S. altissima *** **VOC blend**	**Induced VOC (ng cm**^**-2**^**) ± SE**	**Induced VOC (ng cm**^**-2**^**) ± SE**	***t*-statistic (*P*-value)**	**Induced VOC (ng cm**^**-2**^**) ± SE**	**Induced VOC (ng cm**^**-2**^**) ± SE**	***t*-statistic (*P*-value)**
(*Z*)-3-hexen1ol	1.00 ± 0.03	0.32 ± 0.17	1.62 (0.12)	0.84 ± 0.22	0.04 ± 0.35	1.95 (0.07)
α-pinene	9.29 ± 2.8	2.79 ± 0.95	2.83 (0.01)*	3.76 ± 0.93	1.49 ± 0.62	2.76 (0.01)*
Camphene	0.56 ± 0.27	0.13 ± 0.08	1.78 (0.09)	0.11 ± 0.04	0.06 ± 0.04	-
β-pinene	4.45 ± 1.4	1.44 ± 0.49	2.73 (0.01)*	1.12 ± 0.36	0.44 ± 0.21	2.56 (0.02)*
Myrcene	7.42 ± 1.3	5.43 ± 1.7	2.03 (0.06)	1.68 ± 0.31	0.69 ± 0.24	2.88 (0.01)*
(*Z*)-3-Hexenyl acetate	5.43 ± 1.8	3.41 ± 1.4	-	5.53 ± 0.83	0.92 ± 0.83	1.63 (0.12)
Limonene	14.3 ± 2.9	8.76 ± 3.2	2.12 (0.05)*	3.31 ± 0.54	1.53 ± 0.54	2.65 (0.02)*
(*E*)*-*β-ocimene	0.66 ± 0.25	0.39 ± 0.33	-	0.11 ± 0.04	0.05 ± 0.04	-
Linalool	0.22 ± 0.05	0.20 ± 0.094	-	0.09 ± 0.02	0.05 ± 0.02	1.81 (0.09)
Nonatriene^1^	0.93 ± 0.27	0.98 ± 0.46	-	0.16 ± 0.05	0.11 ± 0.05	-
(*Z*)-3-hexenyl isobutyrate	0.07 ± 0.04	0.03 ± 0.018	-	0.09 ± 0.01	0.03 ± 0.01	1.93 (0.07)
(*Z*)-3-hexenyl butyrate	0.07 ± 0.02	0.062 ± 0.023	-	0.03 ± 0.01	0.04 ± 0.01	-
(*E*)-2-hexenyl butyrate	0.09 ± 0.02	0.089 ± 0.036	-	0.04 ± 0.01	0.03 ± 0.01	-
Bornyl acetate	0.89 ± 0.32	0.594 ± 0.33	1.74 (0.09)	0.03 ± 0.01	0.12 ± 0.06	2.2 (0.04)*
(*Z*)-jasmone	0.27 ± 0.14	0.224 ± 0.093	-	0.04 ± 0.03	-0.07 ± 0.03	2.1 (0.04)*
β-caryophyllene	0.84 ± 0.23	0.655 ± 0.24	-	0.28 ± 0.05	0.12 ± 0.05	2.1 (0.05)*
α-humulene	0.05 ± 0.02	0.006 ± 0.038	-	0.03 ± 0.01	-0.03 ± 0.01	2.9 (0.009)*
β-farnescene	0.27 ± 0.07	0.238 ± 0.084	-	0.09 ± 0.01	0.03 ± 0.01	2.6 (0.02)*
GermacreneD	4.65 ± 1.0	3.37 ± 1.1	-	1.35 ± 0.20	0.46 ± 0.20	2.4 (0.03)*
α-farnescene	0.26 ± 0.09	0.349 ± 0.17	-	0.10 +/0.02	0.06 ± 0.03	-
Nerolidol	0.03 ± 0.02	0.10 ± 0.083	-	0.01 ± 0.01	0.01 ± 0.01	-
Tridecatetraene	0.02 ± 0.02	0.05 ± 0.045	-	0.008 ± 0.01	0.02 ± 0.01	-
Indole	0.05 ± 0.02	0.05 ± 0.03	-	0.04 ± 0.02	0.008 ± 0.004	-

^1^(3*E*)-4,8-dimethyl-1,3,7-nonatriene.

Within daytime and nighttime collections, asterisks (*) indicate statistical comparisons (*t*-tests) of volatile constituents that were significantly different (*P* ≤ 0.05; data shown are untransformed, but statistical analyses were performed on square-root transformed data) between plants that were exposed to the *E. solidaginis* emission and untreated control plants. Dashes (−) indicate overlapping standard errors so no t-tests were conducted. All compounds detected and identified in the *S. altissima* volatile blend are included here.

To test whether this observed increase in HIPV production following exposure to the *E. solidaginis* emission represented a specific response from the co-evolved host plant species or a general plant response to the compounds in the fly emission, we also examined the influence of exposure to the *E. solidaginis* emission on volatile production in maize plants (*Z. mays*). As for *S. altissima*, we found no difference between the total volatile production from undamaged maize plants exposed to the emission or undamaged controls; however, here we also found no difference between the volatile blends induced by *H. virescens* feeding damage on exposed or control plants, indicating that the maize plants did not respond to the *E. solidaginis* emission by enhancing HIPV production (Additional file 3: Table S2). Herbivore-damaged maize plants exposed to the *Eurosta* emission produced 89.2 ± 80.3 ng cm^−2^ during the day and unexposed controls produced 143.9 ± 42.9 ng cm^−2^. Nighttime HIPV production from *Eurosta*-exposed plants was 21.2 ± 8.3 ng cm^−2^ and 13.1 ± 6.6 ng cm^−2^ from unexposed control plants. (Day: two-sided *t*-Test, *t* = 0.60, df = 14, *P* = 0.56; Night: two-sided *t*-Test, *t* = −0.77, df = 14, *P* = 0.46).

### Feeding assays

**Figure 2:**
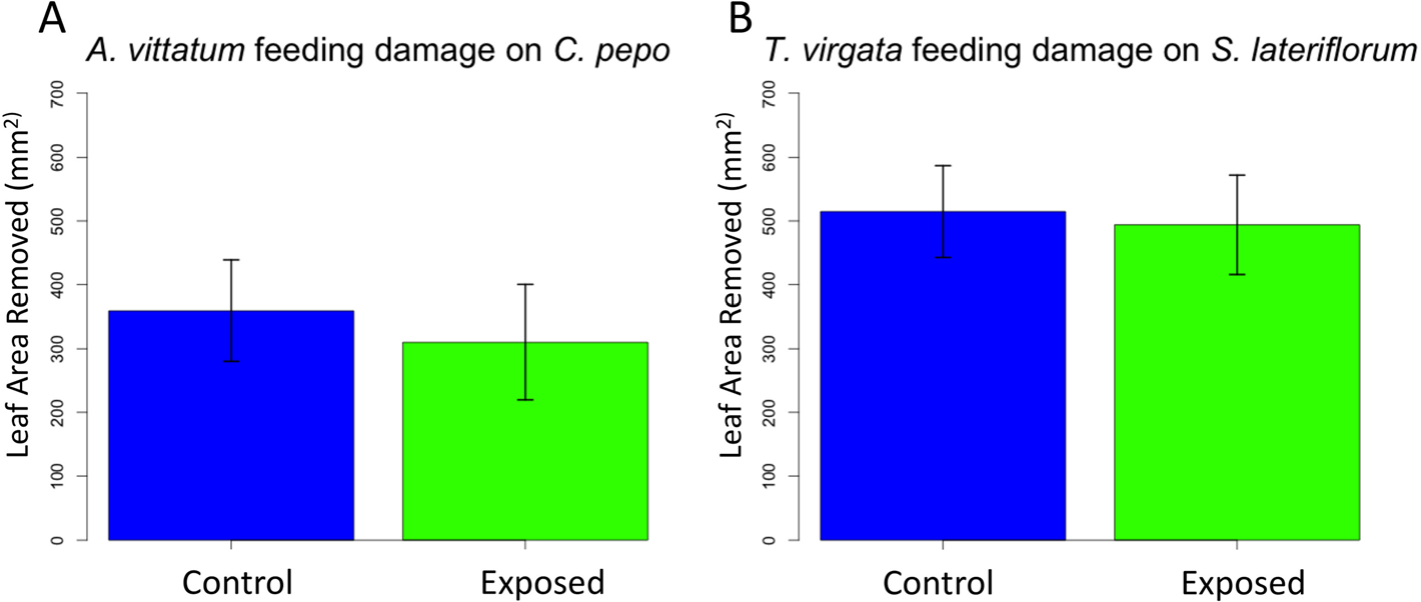
**Herbivore feeding damage on plant species exposed to the *E. solidaginis *emission or not. (A)** Amount of leaf tissue removed by *Acalymma vittatum* on *Cucurbita pepo* and **(B)***T. virgata* feeding on *S. lateriflorum* exposed to the *E. solidaginis* emission or unexposed controls.

To test the hypothesis that the presence of the *E. solidaginis* emission might directly deter insect feeding on exposed plants, we conducted feeding assays with the specialist beetle herbivore *A. vittatum* feeding on exposed and unexposed *C. pepo* plants. *Cucurbita pepo* is only distantly related to *S. altissima* and has no apparent association with *E. solidaginis*. We also performed a similar feeding assay with *T. virgata* feeding on exposed and unexposed *Symphyotrichum lateriflorum. Trirhabda virgata* was employed in our previous study of plant responses to the *E. solidaginis* emission and was found to consume less leaf tissue on emission-exposed *S. altissima*[15]. This species naturally feeds upon *Solidago* and a few closely related genera, including *Symphyotrichum*[37-39]. *Symphyotrichum lateriflorum* is a suitable host plant species for *T. virgata* but not for *E. solidaginis;* therefore, we predicted that exposure to the *E. solidaginis* emission would not enhance *Symphyotrichum lateriflorum* defenses or deter *T. virgata* feeding. We found no significant difference in the total amount of leaf tissue consumed by *A. vittatum* beetles on exposed or control *C. pepo* (Figure 2A, two-sided *t*-test, *t*_18_ = 0.41, *P* =0.69) or *T. virgata* feeding on exposed or control *Symphyotrichum lateriflorum* (Figure 2B, two-sided *t*-test, *t*_12_ = 0.16, *P* =0.87), suggesting that these plant species, which again do not appear to have co-evolutionary history with *E. solidaginis*, did not alter their defenses in response to its emission and the emission did not directly deter herbivore feeding.

## Discussion

We previously reported that *S. altissima* plants perceive the volatile emission produced by male *E. solidaginis* flies and respond by enhancing their anti-herbivore defenses [15]. The findings presented here provide additional physiological evidence of this phenomenon and reveal that a specific downstream anti-herbivore defense—herbivore-induced volatile production—is primed by exposure to the emission. Although the ecological significance of HIPV in this system has not been explored, it is likely that *S. altissima* volatile emissions play a role in plant defense against insect herbivores [36], as has been found for numerous plant species [24-33,40].

Exposure to the emission of male *E. solidaginis* enhanced daytime herbivore-induced volatile production by roughly 44% and nighttime production by roughly 68%. These substantial differences in HIPV production would appear to provide a strong signal for members of the associated arthropod community, possibly including foraging predators and parasitoids. *Eurytoma* parasitoids of *E. solidaginis* are active during the day (personal observation, [41]), but we are unaware of any efforts to characterize their activity levels at night. Given the considerably stronger induction of nighttime volatiles, it seems reasonable to hypothesize that night-active natural enemies may be able to exploit these cues.

A previous study found that primed maize plants, which released more concentrated HIPV emissions preferentially attracted natural enemies compared to unprimed control plants [42]. Some of the compounds emitted in higher concentrations by damaged, *E. solidaginis*-emission-exposed *S. altissima* have previously been linked to defensive roles against insect herbivores in other systems. Green-leaf volatiles (GLV) and terpenes, for example, provide important signals for parasitoid and predator attraction, herbivore repellence, and reduced herbivore performance [30,31,40,43-45]. Additionally, some of the sesquiterpenes primed in this study, including β-farnescene, β-caryophyllene and germacrene D, were also emitted in higher quantities by primed poplar trees and/or maize plants exposed to HIPV [11,46].

In contrast to the *S. altissima* HIPV response following *H. virescens* attack, we observed no similar increase in HIPV production when the same generalist caterpillar species attacked emission-exposed maize plants. These contrasting results support our hypothesis that *S. altissima* plants exhibit an evolved ability to perceive and respond to the emission of its closely associated herbivore *E. solidaginis*. It seems likely that other plant species may also have evolved the ability to detect the pheromones of their herbivores, but we hypothesize that this adaption is most likely to have developed in closely co-evolved plant-insect interactions, likely with monophagous or narrowly oligophagous herbivore species that have a strong influence on host-plant fitness [15].

In our previous work, we observed that both larvae and adults of *T. virgata* consumed less leaf tissue from *S. altissima* plants exposed to the *E. solidaginis* emission compared to control plants [15]. In the same study, we also observed a general reduction of herbivory on the emission-exposed plants in our field experiment, these results are consistent with our hypothesis that the reduced feeding on emission-exposed *S. altissima* plants was the result of an evolved response by *S. altissima* to its specialist herbivore *E. solidaginis*. In the current study, we observed no difference in the feeding of *A. vittatum* on their preferred host plant species *C. pepo,* (which like maize has no obvious relationship to *E. solidaginis*) with and without exposure to the volatile emission of the fly. We also found no difference in feeding damage by *T. virgata* on emission-exposed or control *S. lateriflorum.* This latter result is of particular interest because *Symphyotrichum lateriflorum* is a close relative of *S. altissima* that is a suitable host plant for *T. virgata* but not *E. solidaginis*[37-39]. These results thus strongly indicate that insect herbivores, in this case two herbivorous chrysomelid species, are not directly deterred by the *E. solidaginis* emission.

## Conclusion

The findings presented here provide further support for our hypothesis that *S. altissima* plants can perceive and respond to the putative sex attractant of *E. solidaginis.* In contrast, we found no evidence that the *E. solidaginis* emission directly deters insect feeding. Furthermore, the enhancement of HIPV induction in emission-exposed *S. altissima* plants observed here complements our previous finding that JA induction by herbivory is enhanced in *S. altissima* plants given prior exposure to the fly emission [15], providing additional evidence that reduced herbivory on *S. altissima* plants following exposure to the volatile emission of *E. solidaginis* indeed reflects an evolved adaptive response of this plant species to an olfactory cue from its closely associated herbivore. We can therefore conclude with greater certainty that this system provides the first example of a novel class of plant-insect interactions mediated by plant perception of insect-derived olfactory cues.

## Methods

### The study system

Adult *E. solidaginis* flies typically emerge in mid-May in Pennsylvania and male flies seek perches on goldenrod plants from which to attract mates [41,47]. We discovered that while perching on plants, the male flies emit large quantities of a putative sex pheromone, attractive to female flies (mean ~70 ± 20 μg 24 h^−1^; [15]). After mating, females begin searching for suitable oviposition sites, often ovipositing into the stem of the same or nearby plants. Reproductive output of *S. altissima* plants suffers significantly from galling by *E. solidaginis*[41]; thus, detecting reliable cues associated with impending attack, such as the male fly emission, could provide plants with an advantage in their defense against *E. solidaginis* attack [15]. *E. solidaginis* eggs hatch within 5–8 days and the larval-induced galls usually become visible within 3 weeks [41].

### Plants

We propagated tall goldenrod (*Solidago altissima*) plants from rhizomes of the 110 clone line and grew them in insect-free, climate-controlled growth chambers (16 h light: 8 h dark; 22°C: 20°C; 65% relative humidity (RH)). Rhizomes for this experiment were grown from *S. altissima* originally collected from a field near State College, PA, USA and washed and stored at 4°C prior to planting. We cut rhizomes of similar diameter into 5 cm segments and planted them in shallow trays with peat-based potting soil (Pro-Mix BX; Premier Horticulture Inc., Quakertown, PA, USA). Two weeks after planting, we transplanted the sprouted ramets into individual pots (16 cm diameter, 16.5 cm tall) using the same type of soil and added 0.5 tsp Osmocote fertilizer (8–45–14 N–P–K, Scotts, Marysville, OH, USA) to each pot. *S. altissima* plants used in experiments were 8 wk old and ~ 35 cm tall.

We grew maize plants (*Zea mays* cv. Delprim) from seed in insect-free, climate-controlled growth chambers (16 h light: 8 h dark; 25°C: 25°C; 65% RH). We germinated seeds in the peat-based potting soil and transplanted seedlings into individual pots approximately 1 wk after germination. At this time, plants received 0.5 tsp of the Osmocote fertilizer. *Z. mays* plants used in experiments were in the 3 leaf stage.

We grew wild gourd (*Cucurbita pepo* var. *texana*) plants from seed in insect-free, climate-controlled growth chambers (16 h light: 8 h dark; 23°C: 21°C; 65% RH). We planted seeds in the peat-based potting soil with 0.5 tsp Osmocote fertilizer. *C. pepo* plants used in this experiment were 3.5 weeks old (4 fully expanded leaves).

We grew calico aster (*Symphyotrichum lateriflorum*) plants from rhizomes in insect-free, climate-controlled growth chambers (16 h light: 8 h dark; 23°C: 21°C; 65% RH). The rhizomes for this experiment were harvested from plants grown from seed (Prairie Moon Nursery, Winona, MN, USA) under these same conditions. Importantly, this seed source is within the natural range *E. solidaginis* and its *Solidago* host plant species [41]. The rhizomes were harvested, washed and stored at 4°C prior to planting. We planted 2 cm segments of rhizome in the peat-based potting soil with 0.5 tsp Osmocote fertilizer. *S. lateriflorum* plants used in this experiment were 4 weeks old with a basal rosette of leaves and ~ 20 cm stalk.

### Insects

We collected adult male *Eurosta solidaginis* after they emerged from overwintering galls that we had collected near State College, PA, USA and stored at −20°C. To induce emergence, we placed the galls in a climate-controlled incubator (16 h light: 8 h dark; 22°C, 20°C; 65% RH) for approximately 3 wk.

We reared tobacco budworm (*Heliothis virescens*) larvae in a climate-controlled incubator (16 h light: 8 h dark; 22°C, 20°C; 65% RH) from purchased eggs (Bio-Serv, Frenchtown, NJ, USA) and fed them an artificial casein-based diet. *H. virescens* used in experiments were fourth-instar larvae and were starved for 24 h at room temperature prior to the experiments. Feeding by *H. virescens* caterpillars was previously found to elicit strong volatile production in *S. altissima* plants [36].

We reared striped cucumber beetles (*Acalymma vittatum*) in a laboratory colony from adults collected near State College, PA, USA and fed them growth-chamber grown cucumber plants. Cucumber beetles used in the experiment were mature adults and were starved for 24 h at room temperature prior to the experiment.

We collected goldenrod leaf beetles (*Trirhabda virgata*) from a natural population near State College, PA, USA. We fed the beetles growth-chamber grown *S. altissima* and then starved them for 24 h at room temperature prior to the experiment. Each plant in the experiment received two adult female and one adult male *T. virgata* beetles.

### Collection of the *E solidaginis* emission

Following our previously described methods, we collected the male *E. solidaginis* emission by aerating newly emerged adult male flies in small glass chambers for 24 h [15]. We pushed filtered house air into the chambers at 0.6 L · min^−1^ and pulled air out of the chambers, over an adsorbent filter containing 45 mg of Super-Q (Alltech Associates, Deerfield, IL, USA) at 0.5 L · min^−1^. We eluted filters using 150 μL of dichloromethane and individual samples were pooled to ensure a uniform concentration of emission for the exposure treatments.

### Emission exposure treatments

Inside individual glass chambers (4-L volume), we exposed *S. altissima, Z. mays, C. pepo,* and *S. lateriflorum* plants to crude extracts of the male *E. solidaginis* emission or a dichloromethane solvent control for 24 h [15]. Chambers rested on a two-piece aluminum and Teflon base supported by the rim of the plant pots. The stem of the plant passed through a hole in the aluminum base and was wrapped with cotton to fill the space between the stem and base. To prevent accumulation of condensation and an unrealistic concentration of the *E. solidaginis* emission from building up, filtered air was pushed into the chambers at 3.0 L · min^−1^ and pulled out at 1.0 L · min^−1^. We allowed plants to acclimate to the chambers for 1 h before beginning the exposure treatment. We applied a 12-h male equivalent (40 μL) of the *E. solidaginis* emission crude extract or dichloromethane to each rubber septa and added two septa to each glass chamber. After 12 hours, we added two fresh emission- or solvent-containing septa to each chamber.

### Volatile collections

Using an automated push-pull volatile collection system (Analytical Research Systems, Gainsville, FL, USA), we collected plant-produced volatile compounds from exposed *S. altissima* and *Z. mays* plants before and after herbivore damage. Volatile collections were conducted in a climate-controlled growth chamber (16 h light: 8 h dark; 22°C, 20°C; 65% RH). During the collection, filtered air was delivered into each chamber at 3.0 L · min^−1^ and pulled out of the chamber through an adsorbent filter (containing 45 mg of Super-Q [Alltech Associates, Deerfield, IL, USA]) at 1.0 L · min^−1^. We collected volatiles for 16 h during photophase (06:00–22:00) and on a separate set of filters for 8 h during scotophase (22:00–06:00). After collecting from undamaged plants for 24 h, we introduced two 4^th^ instar *H. virescens* caterpillars into each chamber and allowed them to feed on the plants for 24 h. During this time, we collected damage-induced volatiles following the same schedule. After 24 h, we removed the insects, harvested the plants, and scanned the leaves to calculate the leaf area.

We eluted the volatile trap filters using 150 μL dichloromethane and added to each sample 5 μL of a standard containing nonyl acetate (80 ng/μL) and *n*-octane (40 ng/μL). We quantified amounts of compounds in samples using an Agilent model 7890A gas chromatograph fitted with a flame ionization detector, using a splitless injector held at 220°C. The column (HP-5, 15 m × 0.25 mm × 0.25 μm film thickness; J&W Scientific, Folsom, CA) was maintained at 35 °C for 30 s, then ramped 2°C min^−1^ to 130°C, and ramped again at 20°C min^−1^ to 220°C. We identified volatile components with gas chromatography (Agilent model 7890A) coupled with a mass spectrometer (Agilent model 5975C) in electron ionization mode comparing retention times and spectra with that of pure compounds. Following quantification, the volatile production for each plant was corrected by the total leaf area for that plant (ng cm^−2^). We corrected the volatile production (ng) by the total leaf area (cm^2^) to account for size variation among plants that might have influenced volatile production. To obtain the leaf area, we destructively sampled plants immediately following the collections. Consequently, we used the same leaf-area value to correct the day and night volatiles (neglecting limited leaf area growth during the collection periods).

### Feeding assays

We conducted insect herbivore feeding assays using *C. pepo* and *S. lateriflorum* exposed to either the *E. solidaginis* emission or a solvent control. We exposed plants to a crude extract of the emission or a dichloromethane solvent control following the procedure described above. After 24 h of exposure, we introduced three *A. vittatum* to each of the *C. pepo* and three *T. virgata* to each of the *Symphyotrichum lateriflorum* allowed them to feed on the plants. After 24 h of feeding, we harvested the plants and the scanned their leaves to determine the total area of leaf tissue consumed.

### Statistical analyses

We analyzed the plant volatile data by calculating the herbivore damage-induced volatiles (ng cm ^-2^) produced by each plant (herbivore-damaged plant volatiles – undamaged plant volatiles) during a given time period. We calculated the induced value for each compound in the volatile blend and summed the values to obtain the total induced volatiles for each plant. To account for potential differences in volatile production due to plant size differences, we corrected the induced volatile values for the total leaf area (cm^2^) of the plant. We transformed the *S. altissima* volatiles using a square-root transformation to meet the assumptions of normality and equal variance. We then compared the herbivore-induced volatiles from the emission-exposed and unexposed plants using a two-sided *t*-test for both *S. altissima* and *Z. mays*. We conducted a principle component analysis for the individual compounds of both the day and night *S. altissima* HIPV and constructed biplots of the results. Based on these biplots as well as the standard errors for each compound, we selected individual compound to test using pair-wise comparisons. A two-sided *t*-test was used to compare the amount of leaf tissue consumed in the *C. pepo* and *Symphyotrichum lateriflorum* feeding assays.

## Competing interests

The authors declare that they have no competing interests.

## Authors’ contributions

AMH, CMDM, JFT, and MCM designed research; AMH and JFT performed research; AMH, CMDM, JFT, and MCM analyzed data; and AMH, CMDM, JFT, and MCM wrote the paper. All authors read and approved the final manuscript.

## Supplementary Material

Additional file 1: Table S1 Volatile organic compounds emitted by undamaged *Solidago altissima* plants. Table showing the individual compounds that make up the volatile blend of undamaged *S. altissima* plants. (VOC; means ± standard error; untransformed data shown). Click here for file

Additional file 2: Figure S1A, S1B Biplots from principle component analyses of *Solidago altissima* herbivore-induced volatiles. (A) Biplot of first two principle components of the photophase herbivore-induced volatiles for *E. solidaginis* emission-exposed and unexposed *S. altissima.* Arrows indicate the weight given to individual compounds. Not all compound labels are shown for legibility. Individual plants are labeled with a character representing the treatment (C = control, E = exposed). (B) Biplot of first two principle components of the scotophase herbivore-induced volatiles for *E. solidaginis* emission-exposed and unexposed *S. altissima.* Click here for file

Additional file 3: Table S2 Day and night *Zea mays* individual herbivore-induced volatile organic compounds. Table showing the individual compounds that make up the volatile blend of herbivore-damaged *Z. mays* plants. (VOC; means ± standard error; untransformed data shown). Herbivore-induced volatiles were calculated by subtracting the undamaged volatile production from the herbivore-damaged volatile production (damaged VOC- undamaged VOC). Negative values indicate these compounds were emitted in lower amounts following herbivore-feeding damage. Click here for file
