# Supplementary material for: The volatile emission of Eurosta solidaginis primes herbivore-induced volatile production in Solidago altissima and does not directly deter insect feeding
Source: BMC Plant Biol. 2014 Jun 19;14:173. doi: 10.1186/1471-2229-14-173 (PMC4071026; doi:10.1186/1471-2229-14-173)
Supplement: Additional file 1: Table S1 — Volatile organic compounds emitted by undamaged Solidago altissima plants. Table showing the individual compounds that make up the volatile blend of undamaged S. altissima plants. (VOC; means ± standard error; untransformed data shown). [file 1471-2229-14-173-S1.docx]

|  | Daytime VOC | | Nighttime VOC | |
| --- | --- | --- | --- | --- |
|  | Exposure treatment | | | |
|  | *Eurosta* | Control | *Eurosta* | Control |
| *S. altissima* Volatile Compounds | Undamaged VOC (ng cm^-2^) + SE | Undamaged VOC (ng cm^-2^) + SE | Undamaged VOC (ng cm^-2^) + SE | Undamaged VOC (ng cm^-2^) + SE |
| (*Z*)-3-hexen1ol | 0.234 + 0.12 | 0.147 + 0.09 | 0.676 + 0.29 | 0.780 + 0.27 |
| α-pinene | 0.231 + 0.09 | 0.705 + 0.31 | 0.000 + 0.00 | 0.027 + 0.02 |
| Camphene | 0.090 + 0.05 | 0.142 + 0.08 | 0.093 + 0.05 | 0.059 + 0.02 |
| β-pinene | 0.098 + 0.04 | 0.317 + 0.13 | 0.000 + 0.00 | 0.006 + 0.01 |
| Myrcene | 0.177 + 0.09 | 0.417+ 0.16 | 0.000 + 0.00 | 0.009 + 0.01 |
| (*Z*)-3-Hexenyl acetate | 0.165 + 0.05 | 0.243 + 0.09 | 1.814 + 0.81 | 2.282 + 0.74 |
| Limonene | 0.458 + 0.23 | 1.091 + 0.40 | 0.000 + 0.00 | 0.024 + 0.02 |
| (*E)-*β-ocimene | 0.467 + 0.09 | 0.440 + 0.13 | 0.180 + 0.06 | 0.115 + 0.04 |
| Linalool | 0.048 + 0.02 | 0.046 + 0.02 | 0.004 + 0.00 | 0.000 + 0.00 |
| Nonatriene | 0.222 + 0.08 | 0.533 + 0.27 | 0.004 + 0.00 | 0.000 + 0.00 |
| (*Z*)-3-hexenyl isobutyrate | 0.030 + 0.01 | 0.017 + 0.02 | 0.000 + 0.00 | 0.000 + 0.00 |
| (*Z*)-3-hexenyl butyrate | 0.014 + 0.01 | 0.023 + 0.01 | 0.000 + 0.00 | 0.006 + 0.01 |
| (*E*)-2-hexenyl butyrate | 0.028 + 0.01 | 0.030 + 0.01 | 0.000 + 0.00 | 0.006 + 0.01 |
| Bornyl acetate | 0.011 + 0.01 | 0.098 + 0.04 | 0.000 + 0.00 | 0.009 + 0.01 |
| (*Z*)-jasmone | 0.055 + 0.03 | 0.087 + 0.03 | 0.055 + 0.02 | 0.117 + 0.03 |
| Caryophyllene | 0.041 + 0.02 | 0.076 + 0.04 | 0.000 + 0.00 | 0.000 + 0.00 |
| α-humulene | 0.110 + 0.06 | 0.089 + 0.03 | 0.011 + 0.01 | 0.036 + 0.01 |
| β-farnescene | 0.047 + 0.02 | 0.050 + 0.02 | 0.000 + 0.00 | 0.000 + 0.00 |
| GermacreneD | 0.095 + 0.03 | 0.262 + 0.17 | 0.055 + 0.02 | 0.104 + 0.02 |
| α-farnescene | 0.052 + 0.02 | 0.117 + 0.04 | 0.000 + 0.00 | 0.000 + 0.00 |
| Nerolidol | 0.025 + 0.01 | 0.053 + 0.03 | 0.000 + 0.00 | 0.000 + 0.00 |
| Tridecatetraene | 0.051 + 0.03 | 0.063 + 0.03 | 0.000 + 0.00 | 0.000 + 0.00 |
| Indole | 0.012 + 0.01 | 0.017 + 0.01 | 0.000 + 0.00 | 0.000 + 0.00 |
