# Supplementary figures and images for: The volatile emission of Eurosta solidaginis primes herbivore-induced volatile production in Solidago altissima and does not directly deter insect feeding
Source: BMC Plant Biol. 2014 Jun 19;14:173. doi: 10.1186/1471-2229-14-173 (PMC4071026; doi:10.1186/1471-2229-14-173)

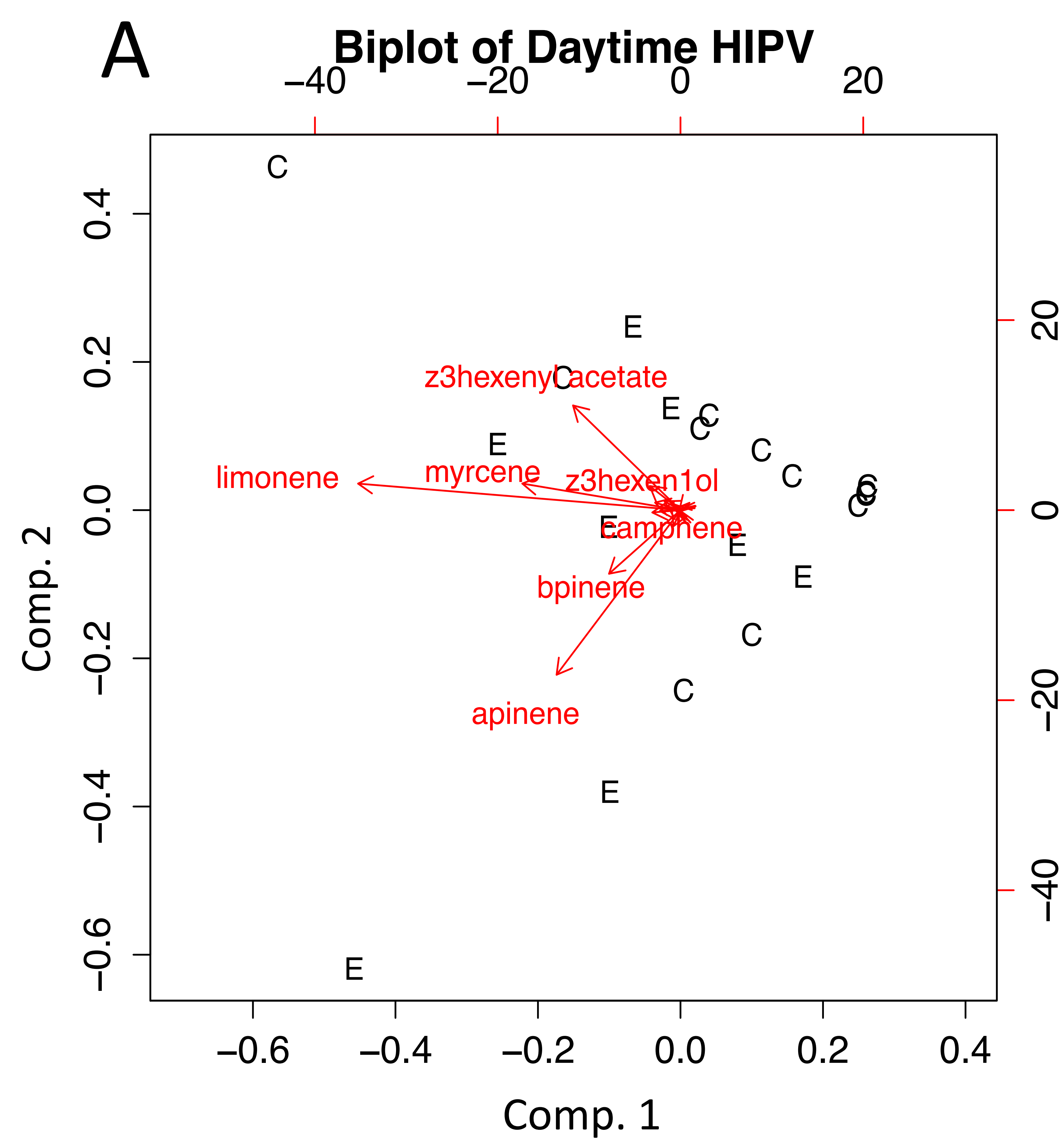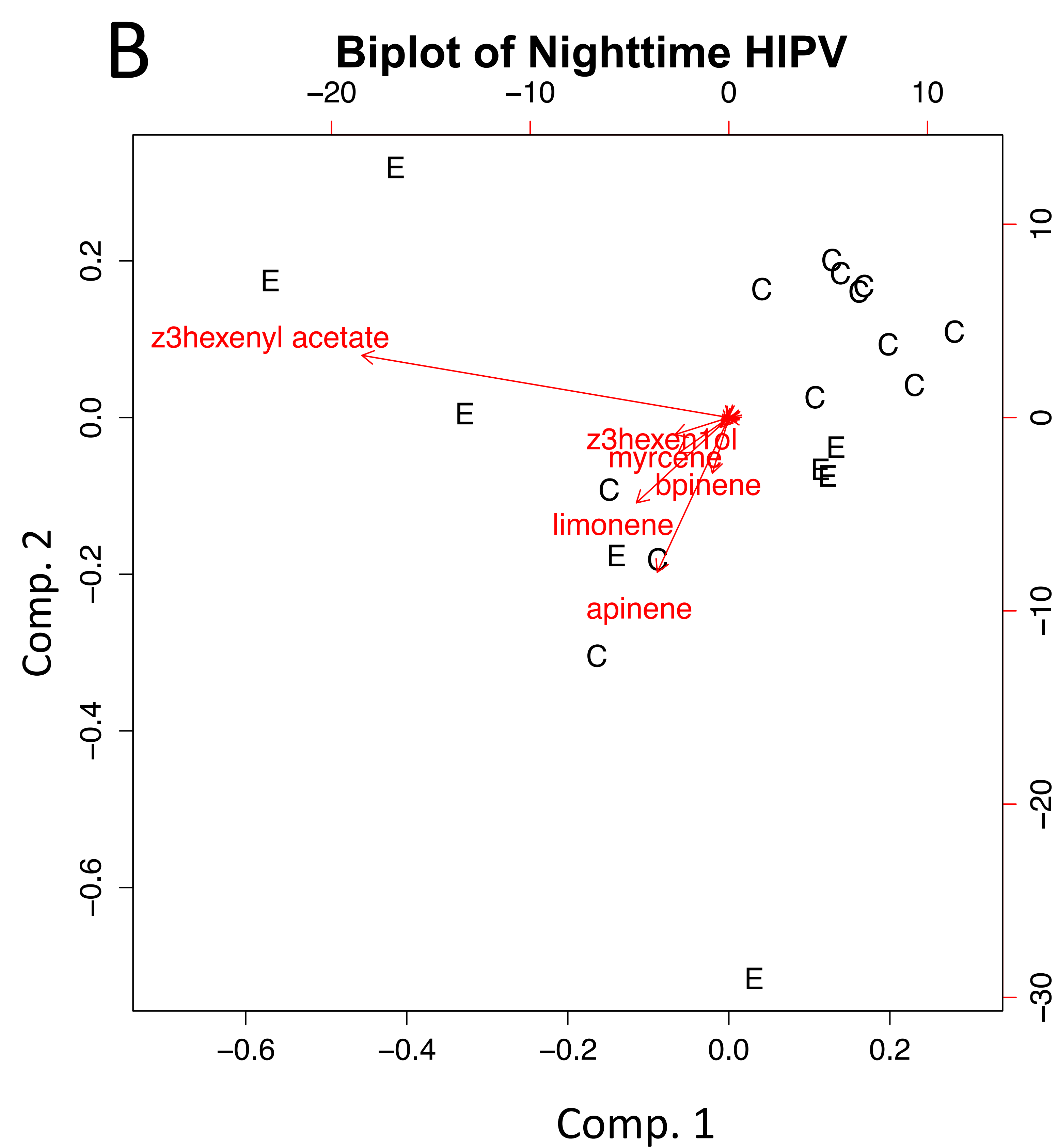

Supplement: Additional file 2: Figure S1A, S1B — Biplots from principle component analyses of Solidago altissima herbivore-induced volatiles. (A) Biplot of first two principle components of the photophase herbivore-induced volatiles for E. solidaginis emission-exposed and unexposed S. altissima. Arrows indicate the weight given to individual compounds. Not all compound labels are shown for legibility. Individual plants are labeled with a character representing the treatment (C = control, E = exposed). (B) Biplot of first two principle components of the scotophase herbivore-induced volatiles for E. solidaginis emission-exposed and unexposed S. altissima. [file 1471-2229-14-173-S2.pdf]
