# Supplementary material for: The volatile emission of Eurosta solidaginis primes herbivore-induced volatile production in Solidago altissima and does not directly deter insect feeding
Source: BMC Plant Biol. 2014 Jun 19;14:173. doi: 10.1186/1471-2229-14-173 (PMC4071026; doi:10.1186/1471-2229-14-173)
Supplement: Additional file 3: Table S2 — Day and night Zea mays individual herbivore-induced volatile organic compounds. Table showing the individual compounds that make up the volatile blend of herbivore-damaged Z. mays plants. (VOC; means ± standard error; untransformed data shown). Herbivore-induced volatiles were calculated by subtracting the undamaged volatile production from the herbivore-damaged volatile production (damaged VOC- undamaged VOC). Negative values indicate these compounds were emitted in lower amounts following herbivore-feeding damage. [file 1471-2229-14-173-S3.docx]

|  | Daytime HIPV | | Nighttime HIPV | |
| --- | --- | --- | --- | --- |
|  | Exposure treatment | | | |
|  | *Eurosta* | Control | *Eurosta* | Control |
| *Z. mays* Volatile Compounds | Induced VOC (ng cm^-2^) + SE | Induced VOC (ng cm^-2^) + SE | Induced VOC (ng cm^-2^) + SE | Induced VOC (ng cm^-2^) + SE |
| (*Z*)-3-hexen1ol | 0.77 + 0.55 | 1.03 + 0.73 | 0.00 + 0.00 | 0.00 + 0.00 |
| Unknown 1 | 8.17 + 4.01 | 6.34 + 5.96 | 0.00 + 0.00 | 0.00 + 0.00 |
| Unknown 2 | 4.54 + 3.79 | 3.07 + 5.10 | 0.00 + 0.00 | 0.00 + 0.00 |
| Unknown 3 | 8.66 + 5.88 | 16.08 + 15.5 | 0.00 + 0.00 | 0.00 + 0.00 |
| Myrcene | -3.59 + 2.17 | -1.59 + 4.24 | 0.00 + 0.00 | 0.00 + 0.00 |
| (*Z*)-3-Hexenyl acetate | 3.48 + 1.24 | 5.20 + 2.38 | 3.71 + 2.05 | 3.79 + 3.72 |
| Limonene | -0.08 + 0.10 | 0.23 + 0.15 | -0.45 + 0.48 | -0.48 + 0.74 |
| (*E)-*β-ocimene | -0.02 + 0.15 | -0.02 + 0.16 | 0.36 + 0.33 | 0.25 + 0.61 |
| Linalool | 1.04 + 0.52 | 2.73 + 1.20 | 20.96 + 13.2 | 41.05 + 34.5 |
| Nonatriene | 0.78 + 0.38 | 1.45 + 0.55 | 9.53 + 5.26 | 12.73 + 10.9 |
| (*Z*)-3-hexenyl isobutyrate | -0.15 + 0.22 | -0.10 + 0.23 | -1.04 + 1.81 | -2.43 + 4.07 |
| (*Z*)-3-hexenyl butyrate | -0.22 + 0.15 | 0.00 + 0.00 | -0.18 + 0.30 | 0.02 + 1.01 |
| (*E*)-2-hexenyl butyrate | -0.22 + 0.15 | 0.00 + 0.00 | 0.40 + 0.17 | 0.70 + 0.53 |
| Bornyl acetate | -0.28 + 0.29 | 0.00 + 0.00 | -0.17 + 0.18 | 0.02 + 0.44 |
| (*Z*)-jasmone | -0.08 + 0.20 | -0.05 + 0.14 | -0.97 + 0.71 | -0.56 + 1.06 |
| Caryophyllene | 0.96 + 0.65 | 1.76 + 0.72 | 12.82 + 9.31 | 12.33 + 10.5 |
| α-humulene | 1.77 + 0.85 | 3.62 + 1.29 | 20.83 + 14.78 | 25.03 + 25.7 |
| β-farnescene | 0.03 + 0.03 | 0.05 + 0.05 | -1.52 + 1.40 | -1.44 + 2.71 |
| Unknown 4 | 7.41 + 6.99 | 9.39 + 10.84 | 0.00 + 0.00 | 0.00 + 0.00 |
| α-farnescene | -2.43 + 1.29 | -2.54 + 3.92 | 0.00 + 0.00 | 0.00 + 0.00 |
| Tridecatetraene | 0.15 + 0.2 | 0.29 + 0.16 | -1.15 + 2.39 | -0.70 + 4.57 |
| Indole | 5.0 + 4.07 | 4.98 + 2.44 | 15.35 + 10.55 | 18.26 + 17.8 |
